# Cardiac Magnetic Resonance in Hypertensive Heart Disease: Time for a New Chapter

**DOI:** 10.3390/diagnostics13010137

**Published:** 2022-12-31

**Authors:** Marija Zdravkovic, Slobodan Klasnja, Maja Popovic, Predrag Djuran, Davor Mrda, Tatjana Ivankovic, Andrea Manojlovic, Goran Koracevic, Dragan Lovic, Viseslav Popadic

**Affiliations:** 1 University Hospital Medical Center Bezanijska Kosa, 11000 Belgrade, Serbia; 2 Faculty of Medicine, Universtity of Belgrade, 11000 Belgrade, Serbia; 3 Department for Cardiovascular Diseases, Clinical Centre of Nis, 18000 Nis, Serbia; 4 Faculty of Medicine, University of Nis, 18000 Nis, Serbia; 5 Clinic for Internal Diseases Inter Medica, 18000 Nis, Serbia; 6 School of Medicine, Singidunum University, 18000 Nis, Serbia

**Keywords:** hypertension, hypertensive heart disease, cardiac magnetic resonance, left ventricle hypertrophy, myocardial fibrosis

## Abstract

Hypertension is one of the most important cardiovascular risk factors, associated with significant morbidity and mortality. Chronic high blood pressure leads to various structural and functional changes in the myocardium. Different sophisticated imaging methods are developed to properly estimate the severity of the disease and to prevent possible complications. Cardiac magnetic resonance can provide a comprehensive assessment of patients with hypertensive heart disease, including accurate and reproducible measurement of left and right ventricle volumes and function, tissue characterization, and scar quantification. It is important in the proper evaluation of different left ventricle hypertrophy patterns to estimate the presence and severity of myocardial fibrosis, as well as to give more information about the benefits of different therapeutic modalities. Hypertensive heart disease often manifests as a subclinical condition, giving exceptional value to cardiac magnetic resonance as an imaging modality capable to detect subtle changes. In this article, we are giving a comprehensive review of all the possibilities of cardiac magnetic resonance in patients with hypertensive heart disease.

## 1. Introduction

Hypertension presents a massive burden for health systems worldwide and is one of the most important cardiovascular risk factors [1]. The incidence of hypertension and its complications, especially in West-Balkan countries, is increasing [2]. More than 70% of people with the first heart attack, stroke, or first episode of heart failure have blood pressure higher than 140/90 mmHg [3]. The impact of untreated and uncontrolled blood pressure is undeniable and can cause complications on different target organs, predominantly kidneys, eyes, brain, peripheral arteries, and also the heart. In general, hypertensive heart disease (HHD) is defined by the presence of left ventricular hypertrophy, left ventricular systolic and diastolic dysfunction, and their clinical manifestations. Different pathophysiological mechanisms inevitably lead to myocardium remodeling and can increase the mortality and cardiac morbidity of patients with hypertension [4]. Myocardial remodeling in these patients is the result of complex myocardial, cellular, and tissue abnormalities leading to changes in the shape, size, and also function of the left ventricle, both diastolic and regional or global systolic [5]. Optimal evaluation of myocardial remodeling is of great importance, not only for making a proper diagnosis but also to follow the impact of the therapy on these patients. Left ventricular hypertrophy, as one of the main characteristics of cardiac remodeling in patients with hypertension, is defined as an abnormal increase in left ventricular mass and is considered to be a result of an adaptation to an increased cardiac workload. The main pathophysiological mechanisms responsible for the progression to LV hypertrophy include not only a response to mechanical stress but also the effect of different neurohormones, growth factors, and cytokines [6]. These complex mechanisms lead to myocyte hypertrophy, as well as myocyte apoptosis, myofibroblast proliferation, and eventually interstitial fibrosis. Although left ventricle hypertrophy and diastolic or systolic dysfunction were marked as the main characteristics of hypertensive heart disease, different novel diagnostic techniques provided more insights into other significant findings in patients with hypertension, especially on diffuse myocardial fibrosis, that can precede the above-mentioned conditions. Myocardial fibrosis in hypertension is originally a part of cardioprotective mechanisms to prevent left ventricular dilatation by increasing ventricular stiffness. This process is diminished in these patients, as it leads to a collagen overproduction, but without a proper protective effect, which gradually leads to the incapability of the ventricle to relax, causing diastolic dysfunction, and heart failure with both preserved (HFpEF), and afterward, reduced ejection fraction (HFrEF) [7]. Currently available imaging modalities to properly estimate these processes in both the subclinical and clinical stages of the disease are limited. Cardiac magnetic resonance is a useful, non-invasive, non-radiating imaging modality with excellent reproducibility and less inter-observer variability that can provide certain, more detailed information on left ventricle volumes and tissue characterization, including scar quantification and the estimation of diffuse myocardial fibrosis. It has a great correlation with 3D echocardiography and speckle-tracking echocardiography, especially in the evaluation of left ventricle volumes and regional systolic function [8]. Although it is not considered a golden standard imaging modality, the usability of cardiac magnetic resonance in hypertensive heart disease is unquestionable.

In this review, we emphasize the emerging diagnostic and prognostic role of cardiac magnetic resonance in patients with HHD.

## 2. Technical Aspects and Possibilities of Cardiac Magnetic Resonance in Patients with Hypertensive Heart Disease

Cardiac magnetic resonance can provide important information in patients with hypertensive heart disease with its high reproducibility, the evaluation of systolic and diastolic dysfunction, and easier and faster evaluation of treatment effects. It is of great value in fibrosis assessment, ischemia detection, and differentiation of multiple causes of left ventricular hypertrophy [9]. The most important sequences that could provide vital information in patients with hypertensive heart disease are steady-state free precession cine (SSFP), phase contrast sequences, T1 and T2 weighted fast spin-echo and T2 STIR sequences, as well as T1-weighted perfusion and myocardial late gadolinium enhancement sequences [10]. Analysis of three-dimensional myocardial strains with tissue tagging gated to diastole is an important modality in estimating diastolic dysfunction [11]. Novel diagnostic procedures under cardiac magnetic resonance, including myocardial tissue mapping by using the modified Look-Locker inversion-recovery sequence and the estimation of extracellular volume fraction are important in the proper assessment of edema, myocardial infiltration, and local or diffuse myocardial fibrosis. Obtaining all these sequences is essential in the proper evaluation of wall motion, function and velocity, tissue characterization, edema, and fibrosis assessment. Although no specific indications are set for patients with HHD, performing cardiac magnetic resonance could be important not only in the early diagnosis of hypertensive heart disease but also in discovering potential complications and following the effects of different treatment modalities.

## 3. Morphological Changes in Patients with Hypertensive Heart Disease

**Figure 1:**
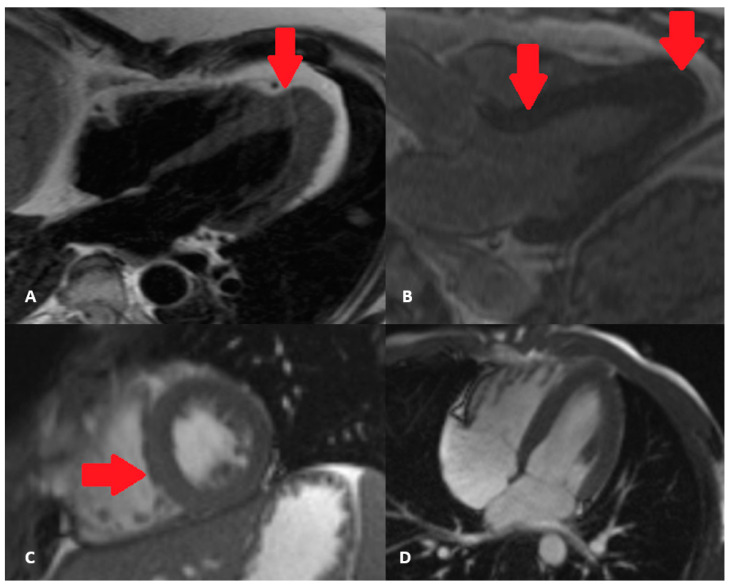
Asymmetric hypertrophic cardiomyopathy–with apical hypertrophy, as marked by arrows (**A**,**B**) versus left ventricular hypertrophy in hypertensive heart disease, concentric type, with predominant hypertrophy of the septum (marked by red arrow) (**C**,**D**) cine sequences.

Long-standing hypertension through increased afterload causes cardiomyocyte hypertrophy and accumulation of interstitial collagen fibers, resulting in left ventricular hypertrophy and diffuse myocardial fibrosis [12]. Left ventricular hypertrophy (LVH) is an independent risk factor for morbidity and mortality in patients with hypertensive heart disease [13,14]. There are different patterns of myocardium hypertrophy, but the clinical impact of all these phenotypes is yet to be evaluated. The most common phenotype is concentric hypertrophy which results in a lower cardiac output due to an increased wall thickening (Figure 1). However, the other types of myocardial hypertrophy should not be ignored, as the worst outcome was noted in patients with dilated (eccentric) hypertrophy and those with thick and dilated hypertrophy [15]. Other conditions in patients with hypertension can affect the hypertrophic pattern, including obesity, diabetes mellitus, and coronary artery disease. It is important to emphasize the fact that diastolic dysfunction and impairment in LV mechanics occur before left ventricular hypertrophy. This is why the estimation of diastolic function and regional systolic function is of great importance. Myocardial fibrosis in these patients can be focal (reparative) or diffuse (interstitial) and is a consequence of the accumulation of type I and type III collagen fibers [16]. By provoking myocardial stiffness, it subsequently leads to changes in ventricular function, myocardial perfusion, and electrical activity. Therefore, it is crucial to identify patients with these abnormalities to prevent the further progression of the disease.

## 4. Comparison of Cardiac Magnetic Resonance with Other Imaging Modalities in Evaluating Hypertensive Heart Disease

Considering the advantages in evaluating patients with hypertensive heart disease, cardiac magnetic resonance, aside from being the gold standard for ventricular function and mass assessment, did not find its place in everyday practice, mostly due to the limited capability of a large number of hospitals to perform it on an everyday basis. Different non-invasive diagnostic modalities have been developed and improved with great correlation to cardiac magnetic resonance in evaluating patients with hypertensive heart disease. In patients with myocardial hypertrophy, ECG showed low sensitivity and specificity, while echocardiography overestimated or underestimated certain clinical forms due to high inter-observer variability [17]. It is shown that echocardiography overdiagnosed LVH in 15% of patients and missed LVH in 14% [18]. However, with the latest echocardiographic techniques, this imaging modality can provide a much wider spectrum of important information. 3D echocardiography showed excellent results in different clinical trials in obtaining measurements of left ventricle size and function in correlation with cardiac magnetic resonance [19]. Speckle-tracking echocardiography can detect subclinical systolic dysfunction and reduced longitudinal strain in patients with hypertensive heart disease [20]. Regarding the differences between various imaging tools in evaluating patients with hypertensive heart disease, cardiac magnetic resonance can offer high reproducibility of measurements, easier and faster evaluation of treatment, intramyocardial function and diastolic dysfunction, fibrosis, and ischemia assessment (Table 1). Ischemia assessment is an important aspect, as cardiac magnetic resonance can provide a highly sensitive non-invasive estimation of ischemia through an adenosine stress-perfusion test [21]. All these parameters give a comprehensive overview of the patient’s clinical condition in one single examination, shortening the time required to make a diagnosis while simultaneously facilitating healthcare expenses.

## 5. Ventricular Volumes and Left Ventricle Geometry Measured by Cardiac Magnetic Disease in Patients with HHD

Pathophysiological mechanisms of hypertensive heart disease involve myocardium remodeling, muscle fibrosis, cardiomyocyte hypertrophy, and hypertrophy of intra-myocardial coronary vasculature. The evolution of the disease is affected by the duration and severity of hypertension, hereditary predisposition, and the effects of cytokines and neurohormonal factors which subsequently lead to systolic and diastolic dysfunction, myocardial fibrosis, or ischemia [22]. Cardiac magnetic resonance is currently the gold standard in estimating right and left ventricular volumes, myocardial mass, non-invasive hemodynamic indexes, as well as accessing the left ventricle geometry [23]. CMR-measured LV mass and cardiac geometry are independently associated with biomarkers of myocardial stretch and injury, including NT-proBNP and hs-Troponin T [24].

**Figure 2:**
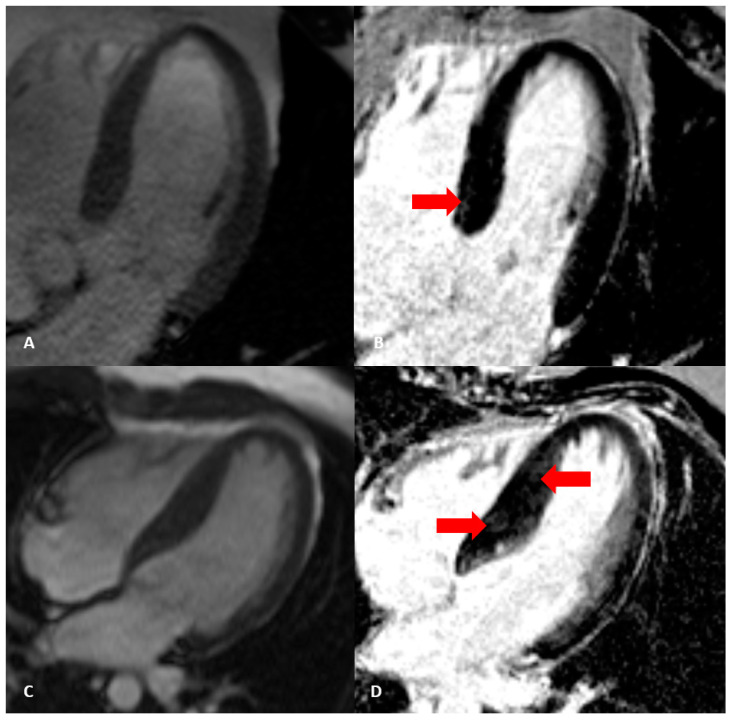
Different cardiac magnetic resonance characteristics in patients with: (**A**,**B**)—hypertensive heart disease (left ventricular hypertrophy with subtle focal LGE marked by red arrow); (**C**,**D**)—hypertrophic cardiomyopathy (characteristic septal LGE marked by red arrow), cine and PSIR LGE sequences.

Optimal evaluation of hypertrophy patterns, especially between hypertrophic cardiomyopathy (HCM) and hypertensive heart disease (HHD), is of great importance considering an increased cardiovascular risk in patients with advanced left ventricle hypertrophy (Figure 2). Additionally, it is important to underline that left ventricle asymmetry and left ventricle end-diastolic wall thickness are poor discriminators between HCM and HHD [25]. A novel imaging technique named myocardial feature tracking is a method similar to speckle-tracking echocardiography which can differentiate between hypertensive heart disease and hypertrophic cardiomyopathy by accessing global longitudinal strain [26]. Although the differences between left ventricle ejection fraction and left ventricle volumes were insignificant in these patients, as presented by Neisius et al., left ventricle mass index, maximum left ventricle wall thickness, late gadolinium enhancement volume, and global native T1 measured by cardiac magnetic resonance were important parameters of HHD to HCM distinction [27].

## 6. Left Ventricle Function Accessed by Cardiac Magnetic Resonance

Global systolic function in the early stages of hypertension is usually preserved. Measuring the intramyocardial strain by cardiac magnetic resonance in these patients is important as this parameter is usually depressed, especially in the septum. Myocardial tissue tagging is a modality that can access longitudinal and circumferential shortening, determine pathophysiological changes, and follow-up left ventricle wall motion changes in patients with hypertensive heart disease. It is shown that in patients with hypertensive heart disease, global circumferential, longitudinal, and radial strain rates were associated with the mean arterial pressure, left ventricular mass index, and age, which is important in preventing the possible consequential decrease in global ejection fraction. Global longitudinal strain is decreased in patients with hypertensive heart disease, regardless of the presence of late gadolinium enhancement phenomenon, and is associated with left ventricular end-diastolic volume, LV ejection fraction, and LV mass index [28]. Certain biomarkers of myocardial fibrosis can be significant in the early stages of hypertensive heart disease before global systolic dysfunction occurs, as they have a good correlation with myocardial strain rates. In patients with decreased longitudinal strain, increased values of serum TIMP-1 level have been detected. This tissue inhibitor of matrix metalloproteinase has been marked as a potential indicator of myocardial fibrosis, not only in hypertensive patients with decreased regional systolic function but also in patients with diastolic dysfunction [29]. Molecular biomarkers of collagen synthesis (PICP and PIIINP) and collagen degradation (CITP and MMP-1) are potential biomarkers of myocardial fibrosis and have significant correlation with left atrial diameter, LV mass, LV posterior wall thickness, LV end-diastolic volume, and longitudinal strain, as important parameters in patients with progressed hypertensive heart disease [30]. Cardiac magnetic resonance derived mitral annular plane systolic excursion (MAPSE) can also be a significant prognostic marker in patients with hypertension. A study by Romano et al. demonstrated that lateral MAPSE was independently associated with mortality across all the subgroups of patients with preserved ejection fraction, even in those without history of previous myocardial infarction [31].

Diastolic dysfunction often develops as a consequence of long-standing hypertension, while atrial dilatation correlates with the severity of hypertension and is associated with increased morbidity and mortality [32]. Quantification of ventricular volume change over time using retrospective gating is an important modality that can estimate atrial filling ratios, peak diastolic filling rate, and time to peak filling [33]. Although it is not used routinely in estimating diastolic dysfunction in patients with hypertensive heart disease, cardiac magnetic resonance could be an important part of stratifying patients with an increased risk to develop a more severe form of the disease.

**Figure 3:**
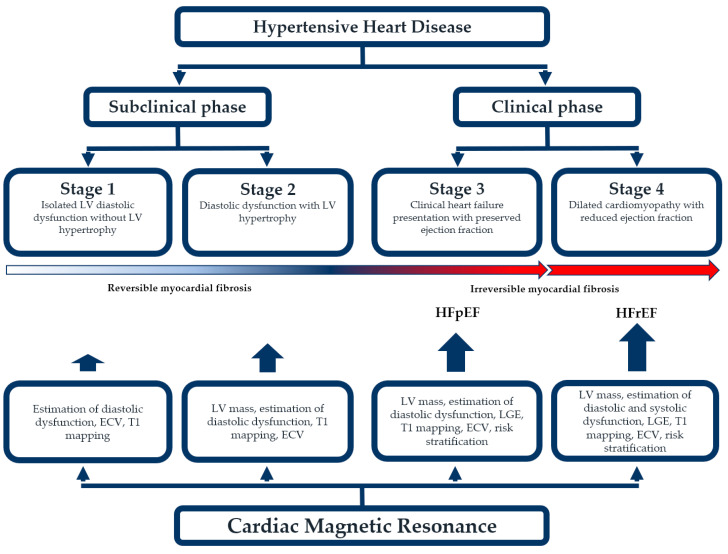
The utility of cardiac magnetic resonance in different stages of hypertensive hearts disease.

Overall, having in mind previously mentioned morphological and functional changes of the myocardium, and based on the clinical impact of hypertension on the heart, hypertensive heart disease can be divided into four categories. The first one consists of isolated left ventricular diastolic dysfunction but without LV hypertrophy. These patients should be closely monitored, and their hypertension controlled as well as possible to prevent further complications. The second one consists of diastolic dysfunction but with concentric LV hypertrophy. This category is usually still a subclinical stage of the disease but with an increased risk of cardiovascular complications, predominantly heart failure. The third stage is a clinical heart failure presentation with preserved ejection fraction (HFpEF). The fourth one implies a dilated cardiomyopathy with reduced ejection fraction [34]. These clinical stages impose a conclusion that diastolic dysfunction in patients with hypertensive heart disease is a more common complication than systolic dysfunction. These patients also have more left ventricular hypertrophy, epicardial coronary artery disease, coronary microvascular dysfunction, and myocardial fibrosis compared to healthy individuals [35]. Cardiac magnetic resonance is more than useful in all these stages, especially in the subclinical phase, which is important in the risk stratification and detection of other underlying pathophysiological mechanisms (Figure 3).

## 7. Tissue Characterization in Hypertensive Heart Disease—Clinical Aspects and Evaluation of Myocardial Fibrosis Using LGE, Myocardial Tissue Mapping and Extracellular Volume Fraction (ECV) Measurement

Expansion of extracellular space in myocardial fibrosis is leading to the accumulation of gadolinium with hyperenhancement on LGE imaging. The presence and degree of focal myocardial fibrosis detected by LGE is an independent and powerful predictor of adverse cardiovascular events in many cardiac conditions [36,37,38]. The presence of late gadolinium enhancement in patients with hypertension, especially those with advanced hypertophy, is usually in a form of patchy non-coronary mid-wall patterns, while patients with co-existant coronary artery disease may have a subendocardial LGE changes. LGE effect in hypertensive heart disease has been detected in up to 50% of symptomatic patients [8]. Even in the group of asymptomatic patients, nonischemic LGE was independently associated with adverse cardiovascular events and was present in up to 18% of the asymptomatic patients. Patients with hypertension and nonischemic LGE were more likely to be men, have diabetes, be current smokers, and have a worse renal function, with higher blood pressure values and taking several antihypertensive medications. These conditions predispose patients to a higher risk of myocardial fibrosis due to the activation of the renin-angiotensin-aldosterone system, β-adrenergic system, and inflammatory and immune pathways. Nonischemic LGE was present more often in patients with greater left ventricular mass, worse multidirectional strain, and elevated circulating markers of myocardial wall stress and myocardial injury, even after the adjustment for the potential confounders [39]. The presence of LGE phenomenon and its association with cardiac remodeling was also observed in patients with nocturnal hypertension, a blood pressure pattern most strongly associated with cardiovascular morbidity and mortality [40]. Considering the fact that myocardial fibrosis is diffuse and interstitial, a proper evaluation through LGE imaging could be a challenging task sometimes. This is the reason why different cardiac magnetic resonance techniques were developed to properly evaluate the extension of myocardial fibrosis, including T1 mapping and extracellular volume fraction.

Diffuse myocardial fibrosis is present in nearly all chronic cardiac conditions. It impairs cardiac function and therefore has a crucial role in the development of heart failure and its outcomes. Estimating the degree of myocardial fibrosis in hypertensive heart disease could be a difficult task having in mind the characteristics of fibrotic changes and the possibilities of different imaging modalities to identify it. Cardiac magnetic resonance, with its superior possibilities in terms of tissue characterization, is a useful tool capable to estimate, not only the presence, but also the extension and severity of fibrosis. A limitation of LGE imaging solely is that it is a qualitative technique, dependent on the difference in signal intensity between normal and fibrotic myocardium. This is why the estimation of diffuse myocardial fibrosis by LGE imaging is inexpedient. The main novel diagnostic modalities used for the evaluation of diffuse myocardial fibrosis are native T1 and post-contrast T1 mapping, and the evaluation of extracellular volume fraction.

The native T1 time can be prolonged due to the expansion of extracellular space caused by myocardial infarction, edema, fibrosis or infiltration, while it can be shortened with the accumulation of fat or iron in the myocardium. One of the main advantages of native T1 time is that it can be measured without the administration of a gadolinium-based contrast agent. Detecting both focal and diffuse myocardial fibrosis is important in predicting chronic heart failure, arrhythmias, and sudden cardiac death. Native T1 time can be prolonged even in the absence of visible LGE phenomenon, pointing out the subclinical phase of diffuse myocardial fibrosis and the potential advantages of novel therapeutic modalities that can prevent the chronic, irreversible stage [41].

Extracellular volume fraction (ECV) is one of the most important tools in estimating myocardial fibrosis. Extracellular volume correlates better than T1 mapping or LGE with histologically determined diffuse myocardial fibrosis. The increased value of ECV is a product of different factors including inflammation, tissue remodeling, atherogenesis, and metabolic disorders. It is shown that ECV has a good correlation with the amount of collagen deposition on myocardial biopsy in various cardiological conditions. The measurement of ECV relies on the values of native T1 and post-contrast T1 time adjusted for blood hematocrit. The pre-contrast T1 value reflects both the intracellular and extracellular compartment, while the post-contrast T1 value primarily reflects the extracellular compartment [42]. The extracellular volume fraction is increased in hypertensive heart disease as hypertension leads to diffuse myocardial fibrosis with the deposition of collagen fibers into the extracellular matrix [43]. It is shown that the presence of both nonischemic LGE and increased extracellular volume is consistently associated with the worst cardiac remodeling pattern, the highest concentration of circulating markers of wall stress and myocardial injury, largest LV mass, increased left atrial volume, and the worst multidirectional strain [44]. ECV can also be important in the differential diagnosis of infiltrative cardiac diseases, hypertrophic cardiomyopathy (HCM), and hypertensive heart disease (HHD) [45] (Table 2).

**Figure 4:**
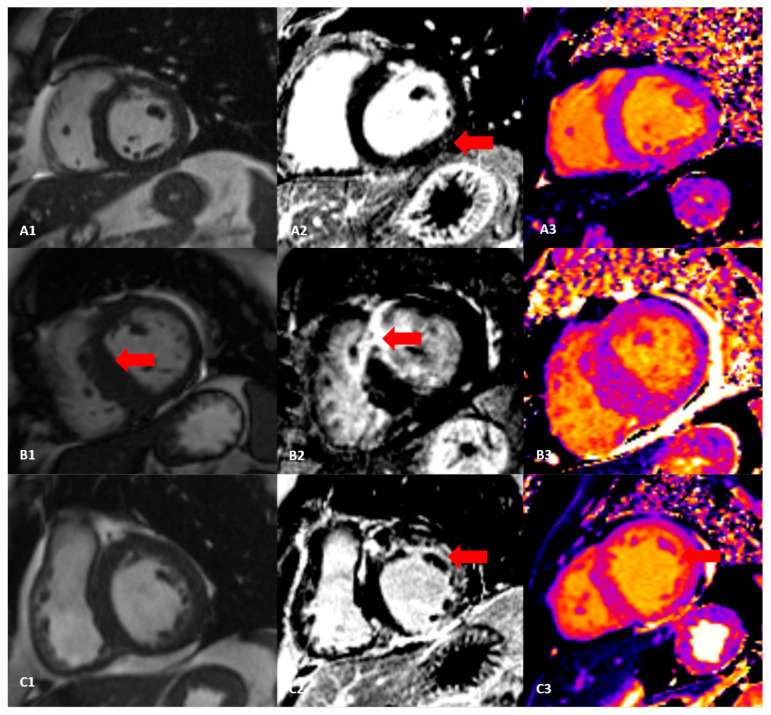
Cine sequences, LGE patterns, and native T1 mapping in patients with hypertensive heart disease, hypertrophic cardiomyopathy, and amyloidosis (**A1**): short-axis cine sequences with hypertrophic septum in a patient with hypertensive heart disease (HHD); (**A2**): subtle focal LGE phenomenon in a patient with HHD (marked by red arrow); (**A3**): T1 mapping showing diffuse borderline values of native T1 time in a patient with HHD; (**B1**): Hypertrophic cardiomyopathy with predominant septal hypertrophy (marked by red arrow); (**B2**): Septal LGE phenomenon in a patient with hypertrophic cardiomyopathy (marked by red arrow); (**B3**): T1 mapping in a patient with HCM revealing prolonged native T1 time (especially in the septum, which is important discriminator between HHD and HCM); (**C1**): cine sequences in short-axis revealing left ventricular hypertrophy in a patient with amyloidosis; (**C2**): diffuse subendocardial LGE phenomenon in a patient with amyloidosis (marked by red arrow); (**C3**): T1 mapping showing prolonged native T1 time in the subendocardium.

Hypertrophic cardiomyopathy can be distinguished from hypertensive heart disease by using ECV and native T1, mostly septal native T1 as the most significant discriminator in these cases [46]. Hinojar et al. found significantly elevated values of ECV in patients with hypertrophic cardiomyopathy than in patients with hypertension [47]. Additionally, patients with amyloidosis have drastically higher levels of ECV than patients with HCM and HHD (Figure 4) [48]. The degree of ECV values and subsequently the extent of fibrosis varies in different hypertensive heart disease phenotypes, being more expressed in patients with pronounced left ventricular hypertrophy. Higher ECV value, increased native T1, and associated reduction in peak systolic circumferential strain, and early diastolic strain rate, are the most significant findings, as presented by Kuruvilla et al. [49]. These patients have an increased risk of coronary artery disease and are more susceptible to developing heart failure, as well as conduction arrhythmias, especially atrial fibrillation [50]. In certain rare clinical scenarios, two different conditions can exist. In these circumstances, it is important to properly estimate the underlying pathophysiological mechanism that dominates the patient’s current clinical condition to enable proper follow-up and forehand therapy.

The most important limitations in measuring ECV by cardiac magnetic resonance are subtle differences between normal patients and patients with hypertensive heart disease. This may limit the clinical usage of T1 mapping and ECV in discovering diffuse myocardial fibrosis, but it is undoubtedly an important imaging modality if applied technically correctly.

## 8. The Role of Cardiac Magnetic Resonance in the Estimation of Coronary Microvascular Dysfunction

In patients with hypertensive heart disease, several structural alterations develop in the coronary microvasculature. Increased medial thickness, reduced maximal cross-sectional area of pre-arterioles and arterioles, and decreased vascular density are the key pathophysiological mechanisms of impaired microcirculatory function [51]. These changes, together with endothelial dysfunction, are the main contributors to the decreased coronary flow reserve in patients with hypertensive heart disease, even in the absence of significant epicardial vessel changes. The combination of microvascular ischemia and myocardial fibrosis is involved in the development of ventricular arrhythmias and increased risk of sudden cardiac death [52]. It has been recently reported that the proportion of sudden cardiac deaths attributable to hypertensive heart disease in the absence of epicardial coronary artery disease has increased [53]. Cardiac magnetic resonance can provide a comprehensive assessment of both epicardial and microvascular coronary circulation through stress perfusion test. Among non-invasive diagnostic modalities, cardiac magnetic resonance has the highest sensitivity and specificity in detecting coronary microvascular dysfunction. Although the qualitative assessment has low sensitivity and specificity, novel quantitative modalities can estimate coronary microvascular dysfunction with much higher accuracy [54]. Perfusion mapping relies on artificial intelligence to obtain perfusion maps, generating conventional images to each image pixel encoding myocardial blood flow, both segmentally and globally. Global myocardial blood flow (MBF) is calculated automatically as an average of all pixels, while myocardial perfusion reserve (MPR) presents the ratio of stress to rest myocardial blood flow. Subendocardial myocardial perfusion reserve has the highest specificity and sensitivity in detecting coronary microvascular dysfunction [55]. It is already shown that patients with impaired coronary microcirculation have a higher incidence of major adverse cardiovascular events [56]. Microvascular dysfunction can be a distinctive subclinical marker of end-organ damage and heart failure in patients with hypertension. In patients with impaired coronary microcirculation in the setting of hypertension, diastolic parameters, global longitudinal strain, and N-terminal pro-B-type natriuretic peptide were independently associated with the degree of impaired myocardial perfusion reserve [57]. It is important to note that these results were obtained in patients without reduced left ventricular ejection fraction or flow-limiting epicardial coronary stenoses. This is why coronary microvascular dysfunction must be investigated in every patient with hypertension and chest pain not associated with obstructive coronary artery disease.

## 9. Advantages of Cardiac Magnetic Resonance in Following the Effects of Anti-Hypertensive Treatment

The possibilities of cardiac magnetic resonance to provide anatomical, physiological, and functional data are of great importance in following the effects of treatment in patients with hypertension. Certain CMR studies have provided enough evidence of the positive role of anti-hypertensive drugs, mainly in terms of left ventricle hypertrophy reduction and changes in cardiac functional parameters [58,59]. Recent technical and software improvements in myocardial tissue characterization enabled a more prevalent use of cardiac magnetic resonance in the studies following the effects of both pharmacological and interventional treatment modalities. Regarding interventional techniques, the advantages of sympathetic renal denervation in the treatment of resistant hypertension have been reported in several cardiac magnetic resonance follow-up studies. Schmidt et al. reported a significant reduction in left ventricular mass and septal wall thickness, while Tahir et al. observed improvements in left ventricular global strain and diastolic function after the renal denervation procedure [60,61]. Mahfoud et al. registered a reduction in the left ventricular mass index, improvement in LV ejection fraction, and circumferential strain [62]. Aside from observing the positive effects of renal denervation on LV mass reduction, diastolic, and systolic function improvement, the impact of this procedure on myocardial fibrosis is still under investigation. However, a study by Doltra et al. demonstrated a significant decrease in ECV value six months after the procedure, independently of blood pressure reduction [63]. This result is not predominantly due to the reversion of myocyte hypertrophy, but also as a result of a reduction in collagen content. The positive effects in terms of a long-standing clinical benefit are yet to be determined.

Numerous studies have evaluated the prognostic value of cardiac magnetic resonance in patients with hypertensive heart disease (as presented in Table 3). Hypertensive heart disease is primarily characterized by left ventricular hypertrophy, left atrial dilatation, myocardial fibrosis, diastolic before systolic dysfunction, and increased incidence of coronary artery disease, both epicardial and microvascular. A comprehensive evaluation of myocardial hypertrophy also allows a proper follow-up of patients on antihypertensive therapy, as the regression of LVH with antihypertensive treatment reduces the risk of stroke, myocardial infarction, and all-cause mortality [27,30]. Morphological and functional changes of the myocardium can be followed through every stage of hypertensive heart disease, even in the subclinical phase, which is important to prevent further complications and development of heart failure with both preserved and reduced ejection fraction. Myocardial strain abnormalities, important in the early detection of myocardial damage, were observed in the majority of the patients. This is significant as it was shown that global longitudinal strain and its deterioration are associated with major adverse cardiovascular events even in patients with asymptomatic hypertensive heart disease [64]. Left atrial enlargement is a common finding in patients with HHD and is an independent factor associated with cardiovascular morbidity and mortality and also the possibility of atrial fibrillation, as presented by Treibel et al. [49]. The signs of incipient interstitial fibrosis are accessible to cardiac magnetic resonance through sophisticated tissue characterization techniques, mainly T1 mapping and ECV, and are also a common finding even in patients with the recent onset of hypertension [65,66,67]. This is important as in the subclinical phase of the disease this type of fibrosis is still reversible with proper and forehand treatment. In the advanced stage with both focal and diffuse fibrosis, myocardial scar quantification can differentiate those patients at high risk for sudden cardiac death, as myocardial scar burden is significantly associated with adverse cardiovascular events, even in patients with preserved ejection fraction [68].

Aside from its accurate and reproducible non-invasive assessment of biventricular function and comprehensive tissue characterization, the extensive application of CMR is hampered by certain limitations. Long examination time, lack of availability and expertise, time-consuming post-processing, and high cost present important technical disadvantages of this imaging modality. Scanning patients with metallic clips, pacemakers, and other non-CMR conditional cardiac devices is not indicated, due to safety reasons. Additionally, paramagnetic contrast agents can not be used in patients with reduced glomerular filtration rate (less than 30 mL/min/1.73 m^2^), which makes this examination limitative in patients with chronic kidney disease. Claustrophobia is also an important patient-related limitation that should be taken into consideration.

## 10. Conclusions

A wide range of clinical and especially subclinical presentations of hypertensive heart disease is often hard to discover, challenging to evaluate properly and incorporate into a proper real-life clinical scenario. The majority of these changes are accessible to the frequently performed diagnostic modalities in an advanced stage of the disease, with an already established high risk of adverse cardiovascular events. Cardiac magnetic resonance is a non-invasive, sophisticated diagnostic tool that can provide more detailed information on left ventricle volumes, tissue characterization, and scar quantification with excellent reproducibility and less inter-observer variability in patients with hypertensive heart disease. It can detect subtle changes in ventricular mass and volume parameters, access overall and regional LV function, and estimate the presence of focal or diffuse myocardial fibrosis. Although it is not recommended as a golden standard in hypertensive heart disease due to its poor availability and relatively low cost–benefit ratio, cardiac magnetic resonance is indicated if there is a severe, progressive left ventricular hypertrophy, an inconclusive echocardiogram or poor acoustic window, and a mismatch between clinical evaluation, ECG, and echocardiography. Additionally, it is of great importance in estimating the positive effects of treatment, as well as an optimal tool in clinical trials. Further studies will bring more information about the benefits of cardiac magnetic resonance in patients with hypertensive heart disease, leading to the more pronounced implementation of this important imaging modality into everyday practice.

## Figures and Tables

**Table 1:** Comparison of imaging modalities used for the estimation of left ventricular hypertrophy.

Characteristics	M-Mode Echo	2D Echo	3D Echo	CMR
Spatial resolution	++++	++++	++	+++
Temporal resolution	++++	++++	+++	+++
Cardiac chambers geometry	++	++	+++	++++
Tissue characterization	-	-	-	+++
Radiation	-	-	-	-
Repeatability	+++	+++	+++	++
Renal failure	-	-	-	+
Mechanical implants	-	-	-	+
Claustrophobia	-	-	-	+
Operator dependent	++	++	++	+
Low availability	-	-	+	++
Cost and resources	+	+	+	+++

**Table 2:** CMR characteristics of various left ventricular hypertrophy patterns.

CMR Characteristics	Hypertensive Heart Disease	Hypertrophic Cardiomyopathy	Amyloidosis	Fabry Disease
LVH	Moderate (<15 mm), concentric or slightly asymmetric IVS/PW <13 mm	Severe, concentric or asymmetric >13 mm (15 mm)	Moderate, concentric LV hypertrophy, RV hypertrophy, IAS hypertrophy, papillary muscle hypertrophy	Moderate, concentric LV hypertrophy, RV hypertrophy, papillary muscle hypertrophy
LVOTO	Rare	Frequent	Rare, possible in severe LV hypertrophy	Rare, possible in severe LV hypertrophy
Severe longitudinal systolic dysfunction	Rare	Frequent	Frequent	Rare
LGE	Less frequent, non-subendocardial, non-specific pattern	Frequent, RV insertion points, intramural “patchy” changes	Diffuse, subendocardial (global or segmental)	Frequent in basal inferolateral segment
Myocardial tissue mapping	Usually normal native T1 and T2 time (or focally increased native T1 time)	Slightly increased native T1 time (especially in septum), usually normal (or slightly increased T2 time)	Diffusely increased native T1 time, normal or slightly increased T2 time	Diffusely decreased global native T1 time, pseudonormalization of native T1 time in basal inferolateral segment
ECV	Normal or slightly increased	Slightly increased	High	Low
Pericardial effusion	Rare	Rare	Frequent	Rare

CMR—cardiac magnetic resonance, LVH—left ventricular hypertrophy, LVOTO—left ventricular outflow tract obstruction, ECV—extacellular volume, RV—right ventricle, IAS—interatrial septum.

**Table 3:** The most significant findings on cardiac magnetic resonance in patients with hypertensive heart disease validated across various studies in recent years.

Authors	Study Sample Size (Patients with HHD)	Gender (Male/Female) (n)	Age (Median)	Control Group (n)	Cardiac Geometry and Volume Assessment	Left Ventricle Function Assessment	Tissue Characterization
Pichler et al. (2020) [30]	36 subjects	30/6	51	No	Increased LV mass and LV mass index	Reduced mean longitudinal and circumferential strain	Increased ECV and ADC (apparent diffusion coefficient)
Kuruvilla et al. (2015) [49]	43 subjects	16/27	59	Yes (22 subjects)	Increased LV mass, increased mass/volume ratio	Reduced peak systolic circumferential strain, reduced early diastolic strain rate	Higher native T1 values, increased ECV
Treibel et al. (2015) [69]	40 subjects	21/19	58.5	Yes (50 subjects)	Left ventricle hypertrophy, increased mass/volume ratio, increased left atrial area index (LAAI), higher LV mass, increased end-systolic and end-diastolic volume	Diastolic dysfunction	Longer native T1 myocardial times, increased ECV in patients with left ventricle hypertrophy
Rodrigues et al. (2016) [65]	88 subjects	50/38	49	Yes (29 subjects)	Increased myocardial cell volume, increased indexed LV mass especially in patients with eccentric LVH	Systolic and diastolic strain impairment, reduced peak systolic circumferential strain values especially in patients with eccentric LVH	Increased native T1 and ECV, most prominent in patients with eccentric LVH
Wu et al. (2017) [66]	30 subjects	10/20	56	Yes (12 subjects)	Increased LV mass and indexed LV mass	Reduced peak circumferential strains at basal and mid-ventricular levels in patients with LVH, reduced early diastolic circumferential strain rate	Higher T1 values, increased ECV, higher ADC (apparent diffusion coefficient) in patients with LVH
Chen et al. (2018) [67]	41 subject	30/11	51	Yes (23 subjects)	Increased indexed LV mass	Reduced peak circumferential, longitudinal and radial strain especially in patients with LVH	Normal T2values, higher T1 values, increased ECV
Neisius et al. (2019) [27]	53 subjects	44/9	60	Yes (64 subjects)	Increased LV mass index	Reduced global longitudinal strain in LGE positive patients, diastolic dysfunction	Increased global native T1 and LGE volume
Mordi et al. (2019) [70]	22 subjects	17/5	67	Yes (28 subjects)	Higher indexed LV mass	Reduced global circumferential strain rate	Higher native T1 and ECV

## Data Availability

Not applicable.
